# 774. Outbreak of Central-Line-Associated Bloodstream Infections (CLABSI) amid the COVID-19 Pandemic Associated with Changes in Central Line Dressing Care Accompanying Changes in Nursing Education, Nursing Documentation, and Dressing Supply Kits

**DOI:** 10.1093/ofid/ofab466.971

**Published:** 2021-12-04

**Authors:** Ahad Azeem, Irene L Newquist, Lesley L Royal, Kimberly S Hemrick, Zachary A Creech, Shiv A Patel, Ajay K Rajan, Gia Thinh D Truong, Faran Ahmad, Marvin J Bittner

**Affiliations:** 1 VA Nebraska-Western Iowa Health Care System/Creighton University School of Medicine, Omaha, Nebraska; 2 VA Nebraska-Western Iowa Health Care System, Omaha, Nebraska; 3 Creighton University School of Medicine, Omaha, Nebraska; 4 Creighton University School of Medicine and VA Nebraska-Western Iowa Health Care System, Omaha, Nebraska

## Abstract

**Background:**

National Healthcare Safety Network (NHSN) data have revealed an increase in CLABSI associated with the COVID-19 pandemic, but data on factors mediating the increase are limited. Our hospital had been free of CLABSI for 18 months, but we encountered an outbreak of 7 CLABSI over a 5-month period beginning in November 2020. This led to an investigation that revealed that some underlying issues were related to COVID-19.

**Methods:**

Infection prevention staff at Omaha’s Veterans Affairs Medical Center interviewed hospital staff and performed a retrospective chart review of patients with CLABSI (based on the NHSN definition) amid the COVID-19 pandemic.

**Results:**

The first case of CLABSI in the outbreak was detected in November 2020. Prior to that, there was no case of CLABSI since April 2019, as shown in the graph. Each case of CLABSI was associated with a different microorganism. Further investigation revealed deviations from our usual practices in central line dressing care. Our response to COVID-19 had included alterations in periodic competency training (including dressing care) for nursing staff as well as the rapid introduction of streamlined inpatient nursing documentation. Previously, dressing kits included chlorhexidine-impregnated dressings; in November, a kit without these dressings was introduced. A weekly audit of dressing care was begun in March 2021. No CLABSI was identified in April 2021.

Types of Microorganisms identified

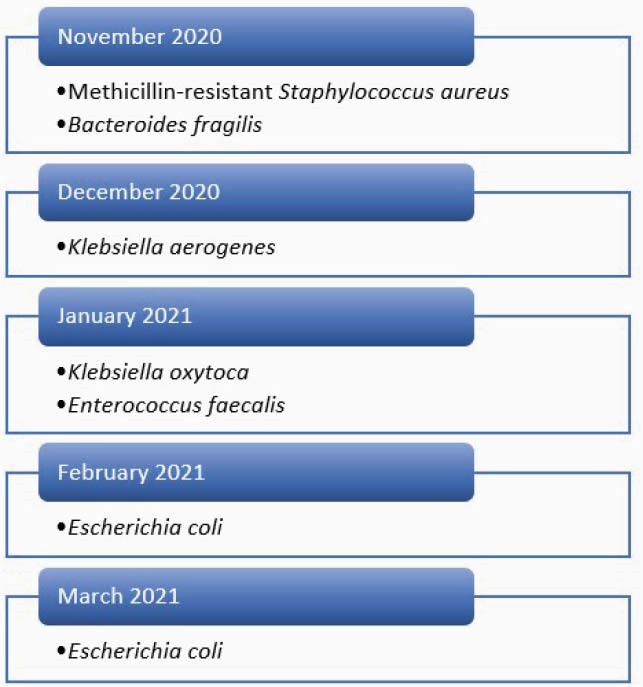

Different types of microorganisms isolated during the CLABSI outbreak each month.

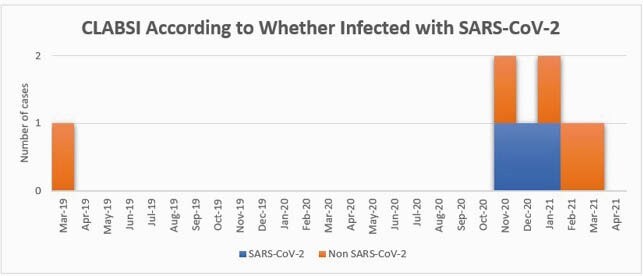

The trend of CLABSI in VA Nebraska-Western Iowa Health Care System

**Conclusion:**

We encountered a CLABSI outbreak associated with deviations from usual central line dressing care. Using the concept of the Swiss cheese model of error prevention, we recognized alterations in three barriers: competency training; thorough documentation; and complete supply kits. The first two of these factors were directly related to our COVID-19 response. Our findings illustrate the relevance of the Swiss cheese model for maintaining a safe healthcare environment.

**Disclosures:**

**Marvin J. Bittner, MD**, **Merck** (Advisor or Review Panel member)**Sanofi Pasteur** (Speaker's Bureau)

